# A complete three-dimensional reconstruction of the myoanatomy of Loricifera: comparative morphology of an adult and a Higgins larva stage

**DOI:** 10.1186/1742-9994-10-19

**Published:** 2013-04-15

**Authors:** Ricardo C Neves, Xavier Bailly, Francesca Leasi, Heinrich Reichert, Martin V Sørensen, Reinhardt M Kristensen

**Affiliations:** 1 Biozentrum, University of Basel, Klingelbergstrasse 50, Basel CH-4056, Switzerland; 2 UPMC–CNRS, FR2424, Station Biologique de Roscoff, Roscoff 29680, France; 3 Academy of Natural Science of Drexel University, 1900 Benjamin Franklin Parkway, Philadelphia, PA, USA; 4 Natural History Museum of Denmark, Universitetsparken 15, Copenhagen Ø, 2100, Denmark

**Keywords:** Confocal microscopy, Phalloidin, Body plan, Phylogeny, Scalidophora, Cycloneuralia

## Abstract

**Introduction:**

Loricifera is a group of small, marine animals, with undetermined phylogenetic relationships within Ecdysozoa (molting protostome animals). Despite their well-known external morphology, data on the internal anatomy of loriciferans are still incomplete. Aiming to increase the knowledge of this enigmatic phylum, we reconstruct for the first time the three-dimensional myoanatomy of loriciferans. Adult *Nanaloricus* sp. and the Higgins larva of *Armorloricus elegans* were investigated with cytochemical labeling techniques and CLSM. We discuss our findings with reference to other loriciferan species and recently established phylogenies.

**Results:**

The somatic musculature of both adult and larval stages is very complex and includes several muscles arranged in three orientations: circular, transverse and longitudinal. In adult *Nanaloricus* sp., the introvert is characterized by a net-like muscular arrangement, which is composed of five thin circular fibers crossed by several (up to 30) thin longitudinal fibers with bifurcated anterior ends. Two sets of muscles surround the pre-pharyngeal armature: 6 buccal tube retractors arranged 3 × 2 in a conical shaped structure, and 8 mouth cone retractors. Additionally, a thick, circular muscle marks the neck region and a putative anal sphincter is the posteriormost myoanatomical feature. In the Higgins larva of *A. elegans*, two circular muscles are distinguished anteriorly in the introvert: a dorsal semicircular fiber and a thin ring muscle. The posteriormost region of the body is characterized by an anal sphincter and a triangular muscle.

**Conclusions:**

Based on the currently available knowledge, the myoanatomical bodyplan of adult loriciferans includes: (i) 8 mouth cone retractors, (ii) a pharynx bulb composed of transversal fibers arranged radially, (iii) circular muscles of the head and neck, (iv) internal muscles of the spinoscalids, (v) longitudinal muscles spanning all body regions, and (vi) transverse (circular) muscles in the abdomen. Concerning the Higgins larva, the muscle subsets assigned to its myoanatomical ground pattern are the (i) longitudinal retractors of the mouth cone, introvert, and abdomen, (ii) abdominal transverse muscles, and (iii) a pharynx bulb composed of transverse, radial fibers. In a comparison with phyla traditionally regarded as phylogenetically close, our data show that the overall myoanatomy of Loricifera is more similar to Kinorhyncha and Nematomorpha than to Priapulida. However, the head musculature of all these groups is very similar, which supports homology of their introverts and head morphology.

## Introduction

Loricifera is a phylum of free living, microscopic animals found exclusively in the marine environment [1]. More than 30 loriciferan species have been found worldwide in coarse marine sands and deep-sea muddy bottom localities at a wide range of depths (e.g., [2-14]). For instance, the phylum’s first described species, *Nanaloricus mysticus*, lives interstitially at 25 m depth off the coast of Roscoff, France, whereas *Pliciloricus hadalis* was collected from red clay at 8260 m in the western Pacific [1,15]. In addition, a large number of new species still await description (see [16,17]). Recently, three oceanographic expeditions revealed the presence of three new species of Loricifera inhabiting a hypersaline, anoxic basin of the Mediterranean Sea [18,19]. Specimens were collected alive, and analyses performed with radioactive tracers, biochemical and ultrastructural methods suggest that these loriciferans complete their life cycle in anoxic conditions, which is unique among metazoans.

Loriciferans possess a minute bilateral body (<500 μm), which is divided into a head (mouth cone and introvert), neck, thorax and abdomen [20]. Distally in the mouth cone, the mouth opening is located at the anterior end of the telescoping mouth tube. Internally, a cuticularized, annulated buccal tube connects the mouth opening to the pharyngeal bulb. Several rings of spine-like scalids of various shapes and sizes protrude from the introvert and neck [20-22]. Adult loriciferans show sexual dimorphism, with males and females differing externally in the number and shape of their scalids. The thorax region lacks external appendages. The abdomen is encased by a cuticular exoskeleton called a lorica, which is composed of several plates or plicae and houses the introvert if retracted [20].

Phylum Loricifera is characterized by a complex life cycle, involving a succession through several larval and post-larval forms to the adult [12,23]. Very little is known about loriciferan embryology, but the cleavage is holoblastic, and the so-called Higgins larva is the first postembryonic stage identified [5]. Although anatomically similar to the adults, this unique larva is mainly distinguished by a pair of flipper- or spine-like, posterior “toes” used for a walking-like locomotion on the substrate (R. Neves, pers. obs.). After several molts the Higgins larva metamorphoses into a post-larval stage (juvenile), which is nearly identical to the adult but lacks gonads (Nanaloricidae; [21]) or is highly modified with reduced scalids (Pliciloricidae; [12]). In some species of family Pliciloricidae, e.g., *Rugiloricus carolinensis-*type, the post-larva is found only inside the Higgins larva and is described as a highly reduced, thin cuticle enclosing an adult form [14]. Generally, the life cycle of species of Pliciloricidae and Urnaloricidae is more complex than that observed in family Nanaloricidae. The former is characterized by the presence of a paedogenetic ghost-larva with internal or external maturation, while the latter is yet to be clarified although several instars of a mega-larva are known to be involved [5,12,24].

The myoanatomy of loriciferans is still poorly understood. So far, there are only incomplete descriptions based on light and transmission electron microscopy observations (see Table 1 and references therein). Ultrastructural studies on the body musculature of adult *Nanaloricus mysticus* and *Pliciloricus enigmaticus* revealed the presence of several circular and longitudinal retractor muscles in the head and in the abdomen [12,20]. In the mouth cone, the musculature is composed by eight longitudinal retractor muscles and 16 radial muscles attached to the furcal base. In the introvert, five inner and 15–24 outer longitudinal retractor muscles are anchored in two to three circular muscles that surround the brain. In addition, six buccal tube retractors seem to be responsible for telescoping the mouth cone and extruding the buccal tube [21]. Since these muscles are located more than 100 μm from the mouth opening, the buccal tube is actually retracted by extremely long muscle attachment fibers. Furthermore, small muscles were found inside the four anteriormost rows of spinoscalids, which is a condition not observed in other scalidophorans, i.e., Kinorhyncha and Priapulida [12,20,25]. Moreover, *N. mysticus* has 10 rings of circular muscle, which are arranged in a position corresponding to the spinoscalids and probably take part in the movement of these individual appendages. In the abdominal region, *P. enigmaticus* possesses 20–22 longitudinal retractor muscles and 11 rings of circular muscles. The scalid and pharyngeal muscles seem to be cross striated, while the longitudinal retractors are obliquely striated [20].

**Table 1:** Data available prior to this study on the musculature of Loricifera

**Species**	**LM/TEM**	**Mouth cone muscles**		**Introvert muscles**			**Abdomen muscles**		**Pharyngeal bulb**	
		**Longitudinal**	**Radial**	**Longitudinal**	**Circular**	**Others**	**Longitudinal**	**Circular**		**Reference**
**(Adults)**										
*Nanaloricus mysticus*	TEM	8 (inside the ridges)	16 (attached to the furcal base)	5 inner and 15–24 outer	2-3 (surrounding the brain)	6 buccal tube retractors; spinoscalid muscles; 10 rings (scalid rows)	(nd)	(nd) ^a)^	myoepithelial, radial musculature	[12,20,21]
Armorloricus elegans*	TEM, LM	(np)	(np)	6 inner and at least 15 outer; 1 dorsal pair spans through the whole body (widely separated)	1 (surrounding the brain)	spinoscalid muscles	1 dorsal pair (same fiber that runs in the introvert)	several diagonal and dorso-ventral muscles	myoepithelial, radial musculature	[2,12,25]
*Pliciloricus enigmaticus*	TEM	8 (inside the ridges)	16 (attached to the furcal base)	5 inner and 15–24 outer	2-3 (surrounding the brain)	spinoscalid muscles; scalid ring muscles^b)^	20-22	11 (ring-like)	myoepithelial, radial musculature	[20]
*Pliciloricus pedicularis*	LM	8 (run through the brain)	−	1 pair large; neighbor retractors present (smaller)	1 (ring-like)	spinoscalid large diagonal muscles	several (located anteriorly)	several transverse muscles; 1 (ring-like, posterior)	3 transversal layers of radial muscles	[10]
*Pliciloricus diva*	LM	8 (run through the brain)	−	1 pair large; neighbor retractors present (smaller)	−^c)^	spinoscalid diagonal muscles; scalid ring muscles	several (located anteriorly)	3 layers of transverse muscles;	(nd)	[11]
*Rugiloricus doliolius*	LM	8 (run through the brain)	−	1 dorsal pair (large)	− ^c)^	spinoscalid muscles; scalid ring muscles	several bundles	many bundles (ring muscles)	(nd)	[16,26]
*Rugiloricus renaudae*	LM	present	−	present	present	spinoscalid muscles	several strings	narrow bundles (transverse)	(nd)	[13]
**(Higgins larva)**										
*Pliciloricus pedicularis*	LM	−	−	−	−	−	several	several (transverse)	muscles arranged in five layers of 3 radial muscles	[10]
*Pliciloricus diva*	LM	−	−	−	−	−	−	−	muscles arranged in five layers of 3 radial muscles	[11]
*Rugiloricus doliolius*	LM	6 short	−	8	− ^c)^	spinoscalid muscles	8 (ventral pair strongest); toe base muscles	7 ring muscles	(nd)	[16,26]

*Description partially based on direct observations of images published in Kristensen [25] and Bang-Berthelsen et al. [12]; (−), not found; (nd), included in figures but not described; (np), images not provided; ^a)^Though a neck circular muscle is described in Bang-Berthelsen et al. [12]; ^b)^Fewer than in *N. mysticus*; ^c)^Though transverse (or ring) muscles are described in the thoracic region.Myoanatomical features of adult and Higgins larva stage observed by light (LM) or transmission electron microscopy (TEM).

Although the ultrastructure of *Armorloricus elegans* was never investigated in detail, data on its myoanatomy are currently available (cf. [12]). In this nanaloricid species, the introvert musculature includes six inner and at least 15 outer longitudinal retractors, and a large circular muscle that surrounds the brain. In addition, a cross section at the level of the second row of the introvert scalids showed the presence of two small muscles inside the base of these spine-like appendages (cf. [25]). In the original description of *A. elegans*, investigation of semithin sections revealed several diagonal and dorso-ventral muscles located in the abdomen and a dorsal pair of retractors that spans the whole body [2].

More recently, the musculature of *Pliciloricus pedicularis*, *Pliciloricus diva*, *Rugiloricus doliolius* and *Rugiloricus renaudae* was investigated by light microscopy [10,11,13,16,26]. Longitudinal retractor muscles are present in the mouth cone (usually 8), introvert (1 pair) and abdomen (several) of all these species, which is similar to the condition found in *P. enigmaticus* and *N. mysticus*. However, the introvert circular muscles were observed only in *P. pedicularis* and *R. renaudae*, and the radial muscles attached to the furcal base of the mouth cone were not found in any of the recently investigated species (for more details see Table 1).

As for the body musculature of the Higgins larva, the available data is scarce and comes solely from light microscopy observations on *P. pedicularis* and *R. doliolius*[10,16]. Both species possess longitudinal and circular muscles in the abdomen region, but the mouth cone and head longitudinal muscles were found only in the latter species (for more details see Table 1). Furthermore, no radial/circular muscles were found in the mouth cone or introvert in any of these two species. In addition, the Higgins larva of *R. doliolius* possesses muscles inside the spinoscalids, as well as, inside the bases of the posterior toes [16].

The pharyngeal musculature of several Loricifera species was investigated by light- and transmission electron microscopy as well (see Table 1; [2,10-12,16,20,27]). In adults of family Pliciloricidae and all loriciferan larvae, the pharyngeal bulb is located at the base of the mouth cone, while in adults of family Nanaloricidae this myoepithelial structure is located below the mouth cone. The pharyngeal bulb of both adult and larval stages is composed of fibers arranged in radial layers and possesses a triangular lumen (see [27]), for a morphological comparison with phylum Tardigrada). Two types of myofibrils are found in the pharyngeal musculature: those attached to the placoids and those attached between the placoids [20].

To date, a complete description of the myoanatomy of Loricifera is lacking, notably because earlier attempts to perform phalloidin stainings always rendered unsatisfactory results [12]. Here, we present a complete three dimensional description of the muscular organization of adult *Nanaloricus* sp. and larval *Armorloricus elegans* based on the labeling of F-actin present in muscles combined with confocal laser scanning microscopy (CLSM). Moreover, we consider the changes that occur in the muscular system at different stages of adult body retraction. The results are compared with earlier data on the myoanatomy of Loricifera (cf. [10-13,16,20,26]) and other scalidophoran phyla, i.e., Kinorhyncha and Priapulida.

## Results

### Gross morphology of the adult *Nanaloricus* sp

**Figure 1:**
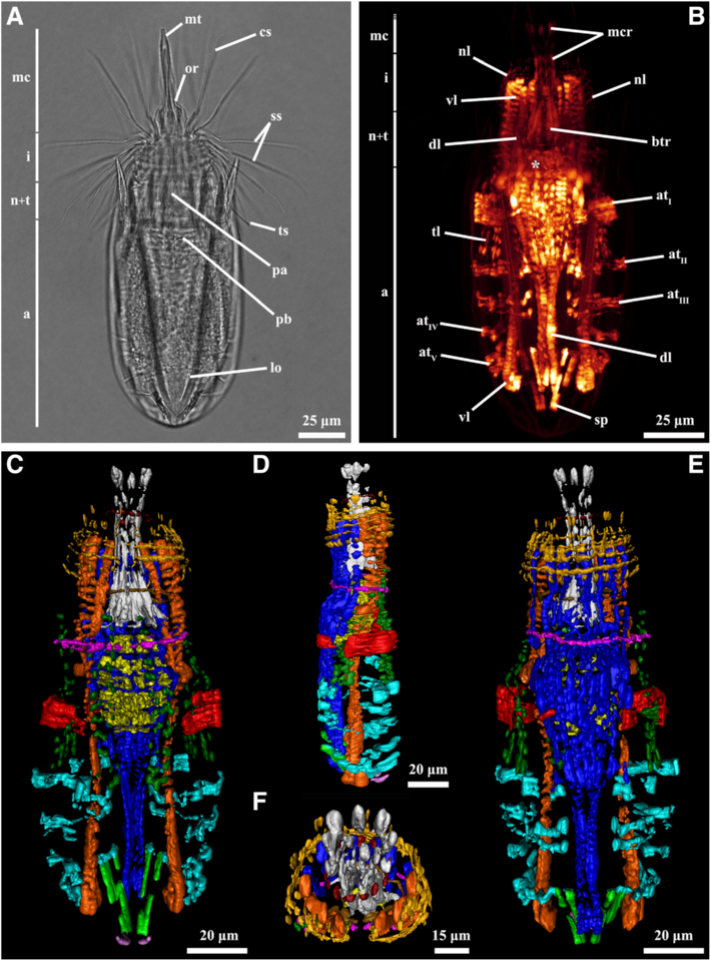
***Nanaloricus *sp. Light micrography of an adult stage (A).** Body musculature visualized by fluorescently-labeled phalloidin staining and CLSM (**B**) and by 3D imaging (**C-F**). Anterior faces upwards in all aspects. **A**: Note the fully extended introvert with several protruding scalids. **B**: Dorsal view. **C**: Ventral view. **D**: Lateral view. **E**: Dorsal view. Note especially the complex arrangement of thin fibers in the head and thoracic region. The prominent pharyngeal bulb musculature is located anteriorly to the abdominal transverse muscle I. **F**: Frontal view of the anteriormost body region. Color code: abdominal transverse muscle I, red; abdominal transverse muscles II-V, light blue; buccal tube retractors and mouth cone retractors, white; dorsal longitudinal retractor muscle, dark blue; introvert anterior ring muscle, dark red; introvert posterior ring muscle, brown; neck circular muscle, purple; net-like thin muscles, dark yellow; pharyngeal bulb musculature, yellow; short posterior longitudinal muscles, green; posteriormost short curved muscles, light purple; thin longitudinal muscles, dark green; ventral longitudinal retractor muscle, orange. Abbreviations: *, pharyngeal bulb musculature; a, abdomen; at_I-V_, abdominal transverse muscles; btr, buccal tube retractors; cs, clavoscalid; dl, dorsal longitudinal retractor muscle; i, introvert; lo, lorica; mc, mouth cone; mcr, mouth cone retractors; mt, mouth tube; n + t, neck and thorax; nc, neck circular muscle; nl, net-like thin muscles; or, oral ridge; pa, pre-pharyngeal apparatus; pb, pharyngeal bulb; ss, spinoscalid; sp, short posterior longitudinal muscles; tl, thin longitudinal muscles; ts, trichoscalid; vl, ventral longitudinal retractor muscle.

**Figure 2:**
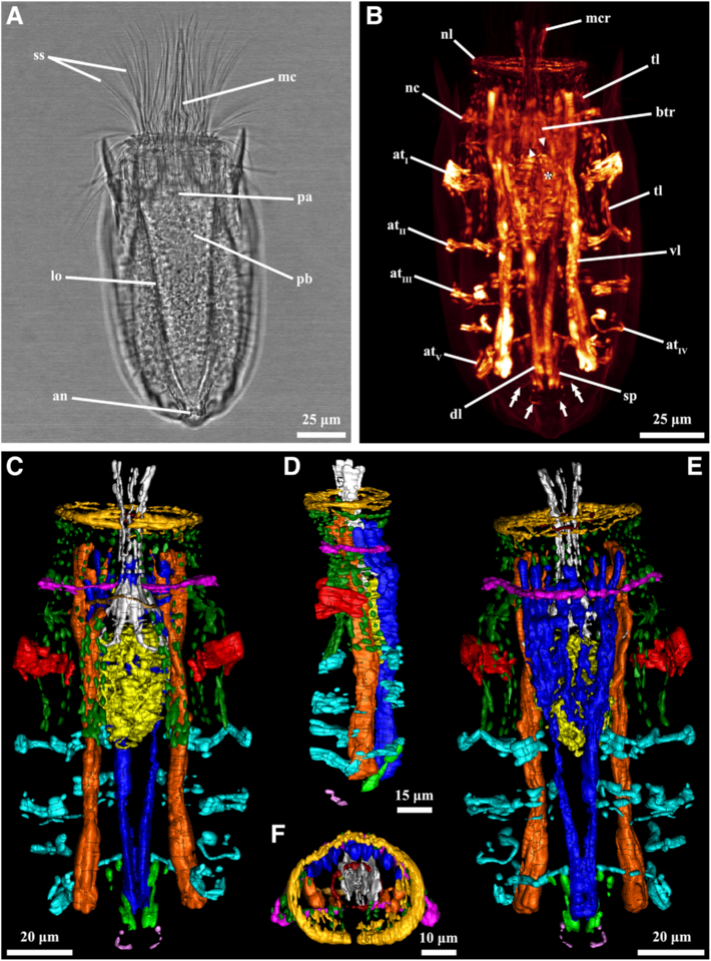
***Nanaloricus *sp. Light micrography of an adult stage (A).** Body musculature visualized by fluorescently-labeled phalloidin staining and CLSM (**B**) and by 3D imaging (**C-F**). Anterior faces upwards in all aspects. **A**: Note the slightly retracted introvert and the several scalids projecting forward. **B**: Dorsal view. **C**: Ventral view. **D**: Lateral view. **E**: Dorsal view. In this specimen the net-like thin muscles in the head are arranged as a thick ring-like structure. Note, as well, that the pharyngeal bulb musculature is located between abdominal transverse muscle I and II. **F**: Frontal view of the anteriormost body region. Color code: abdominal transverse muscle I, red; abdominal transverse muscles II-V, light blue; buccal tube retractors and mouth cone retractors, white; dorsal longitudinal retractor muscle, dark blue; introvert anterior ring muscle, dark red; introvert posterior ring muscle, brown; neck circular muscle, purple; net-like thin muscles, dark yellow; pharyngeal bulb musculature, yellow; short posterior longitudinal muscles, green; posterior short curved muscles, light purple; thin longitudinal muscles, dark green; ventral longitudinal retractor muscle, orange. Abbreviations: *, pharyngeal bulb musculature; an, anus; at_I-V_, abdominal transverse muscles; btr, buccal tube retractors; dl, dorsal longitudinal retractor muscle; lo, lorica; mc, mouth cone; mcr, mouth cone retractors; nc, neck circular muscle; nl, net-like thin muscles; pa, pre-pharyngeal apparatus; pb, pharyngeal bulb; ss, spinoscalid; sp, short posterior longitudinal muscles; tl, thin longitudinal muscles; ts, trichoscalid; vl, ventral longitudinal retractor muscle; arrows, posterior short curved muscles; arrowheads, introvert posterior ring muscle; double arrows, posterior thin fibers.

**Figure 3:**
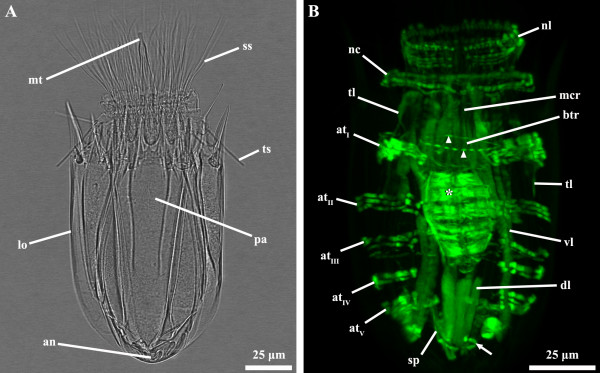
***Nanaloricus *sp. Light micrography of an adult stage (A) and body musculature visualized by fluorescently-labeled phalloidin staining and CLSM (B).** Anterior faces upwards in both aspects. **A**: Note the almost fully retracted introvert and all scalids projecting forward, while the trichoscalids are oriented posteriorly. **B**: Dorsal view. The net-like thin musculature is located very close to the neck circular muscle. Note, as well, that the pharyngeal bulb musculature occupies a medial region within the abdominal cavity. Abbreviations: *, pharyngeal bulb musculature; an, anus; at_I-V_, abdominal transverse muscles; btr, buccal tube retractors; dl, dorsal longitudinal retractor muscle; lo, lorica; mcr, mouth cone retractors; mt, mouth tube; nc, neck circular muscle; nl, net-like thin muscles; pa, pre-pharyngeal apparatus; ss, spinoscalid; sp, short posterior longitudinal muscles; tl, thin longitudinal muscles; ts, trichoscalid; vl, ventral longitudinal retractor muscle; arrow, posterior short curved muscle; arrowheads, introvert posterior ring muscle.

**Figure 4:**
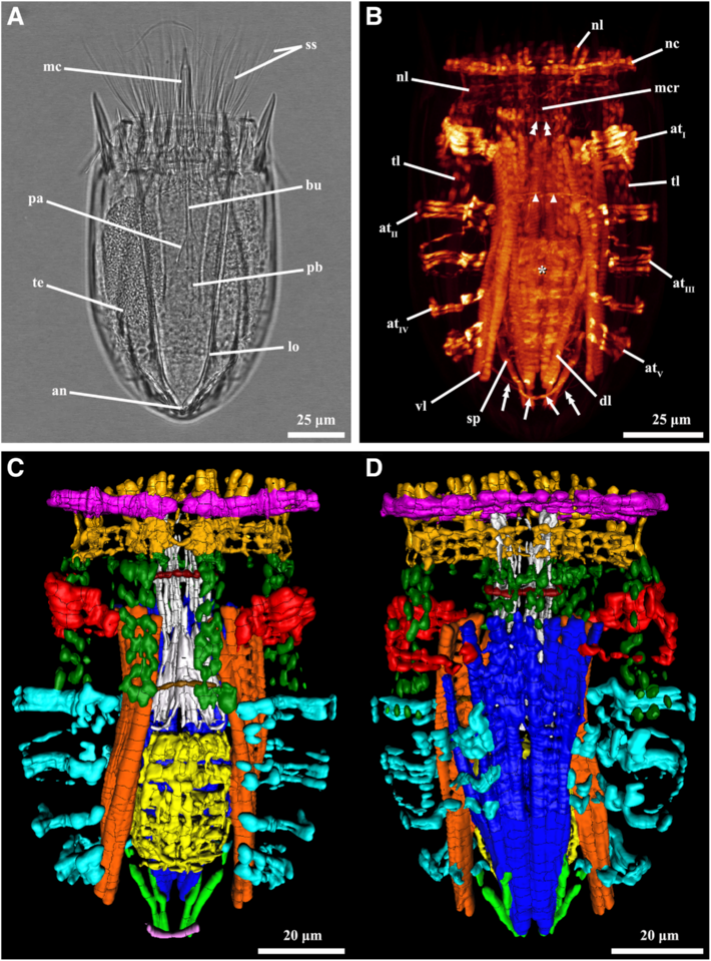
***Nanaloricus *sp. Light micrography of an adult stage (A).** Body musculature visualized by fluorescently-labeled phalloidin staining and CLSM (**B**) and by 3D imaging (**C-D**). Anterior faces upwards in all aspects. **A**: Male specimen with introvert retracted. **B**: Ventral view. **C**: Ventral view. **D**: Dorsal view. In this specimen the neck circular muscle merges the net-like thin muscles in the head. The pharyngeal bulb musculature is located posteriorly within the abdominal cavity. Color code: abdominal transverse muscle I, red; abdominal transverse muscles II-V, light blue; buccal tube retractors and mouth cone retractors, white; buccal tube ring muscle, dark red; dorsal longitudinal retractor muscle, dark blue; introvert anterior ring muscle, dark red; introvert posterior ring muscle, brown; neck circular muscle, purple; net-like thin muscles, dark yellow; pharyngeal bulb musculature, yellow; short posterior longitudinal muscles, green; posterior short curved muscles, light purple; thin longitudinal muscles, dark green; ventral longitudinal retractor muscle, orange. Abbreviations: *, pharyngeal bulb musculature; an, anus; at_I-V_, abdominal transverse muscles; bu, buccal tube; cs, clavoscalid; dl, dorsal longitudinal retractor muscle; lo, lorica; mc, mouth cone; mcr, mouth cone retractors; nc, neck circular muscle; nl, net-like thin muscles; pa, pre-pharyngeal apparatus; pb, pharyngeal bulb; ss, spinoscalid; sp, short posterior longitudinal muscles; te, testis; tl, thin longitudinal muscles; vl, ventral longitudinal retractor muscle; arrows, posterior short curved muscles; arrowheads, introvert posterior ring muscle; double arrows, posterior thin fibers; double arrowheads, buccal tube ring muscle.

**Figure 5:**
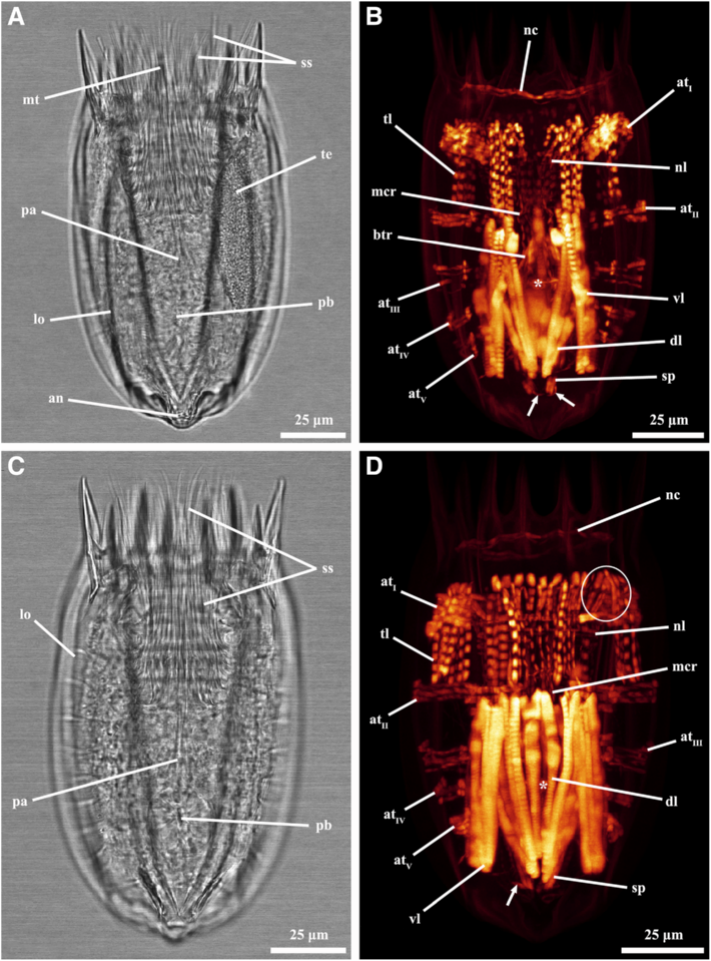
***Nanaloricus *sp. Light micrography of adult stages (A and C) and body musculature visualized by fluorescently-labeled phalloidin staining and CLSM (B and D).** Anterior faces upwards in all aspects. **A**: Same specimens as in **B**. Note that the introvert is fully retracted as in the specimen shown in **C**. **B**: Ventral view. Note that the net-like structure is located posteriorly to the neck circular muscle. **C**: Same specimens as in **D**. **D**: Dorsal view. The circle encloses a few thin longitudinal muscles folding inwards. Abbreviations: *, pharyngeal bulb musculature; an, anus; at_I-V_, abdominal transverse muscles; btr, buccal tube retractors; dl, dorsal longitudinal retractor muscle; lo, lorica; mcr, mouth cone retractors; nc, neck circular muscle; nl, net-like thin muscles; pa, pre-pharyngeal apparatus; ss, spinoscalid; sp, short posterior longitudinal muscles; te, testis; tl, thin longitudinal muscles; vl, ventral longitudinal retractor muscle; arrow, posterior short curved muscle.

The adult specimens investigated in this study resemble *Nanaloricus mysticus* in many respects, but also differ in some details (cf. [1,20]). Even though they obviously are congeners, the examined specimens clearly represent a yet undescribed species, conspecific with the species that was sequenced for the study of Sørensen et al. [28]. The body of *Nanaloricus* sp. is divided into five main regions: mouth cone, introvert, neck, thorax, and abdomen (Figures 1A, 2A, 3A, 4A, 5A,C). The mouth cone is short and surrounded by 8 oral ridges of equal length, which were named oral stylets in the original description of *N. mysticus* (Figure 1A). The distal region of the mouth cone is a cuticular mouth tube ending with a terminal mouth opening (Figures 1A, 3A, 4A, 5A,C). A single ring of clavoscalids and 8 rings of spinoscalids of various shapes and sizes protrude from the head (Figures 1A, 2A, 3A, 4A, 5A,C), while the neck region is characterized by the presence of 15 trichoscalids (Figures 1A, 3A). The thoracic region is usually concealed inside the cuticular exoskeleton called the lorica and lacks appendages. The abdomen is encased by the lorica, which is composed of 6 plates with a honeycomb sculpturing. The anus-gonopore complex is positioned postero-dorsally. Internally, the head/thoracic region is characterized by the long buccal tube and the pre-pharyngeal armature, which is composed of three internal furcae (Figures 1A, 2A, 3A, 4A, 5A,C). The pre-pharyngeal armature supports the buccal tube and is located immediately anterior to the pharyngeal bulb.

### The myoanatomy of adult *Nanaloricus* sp

**Figure 6:**
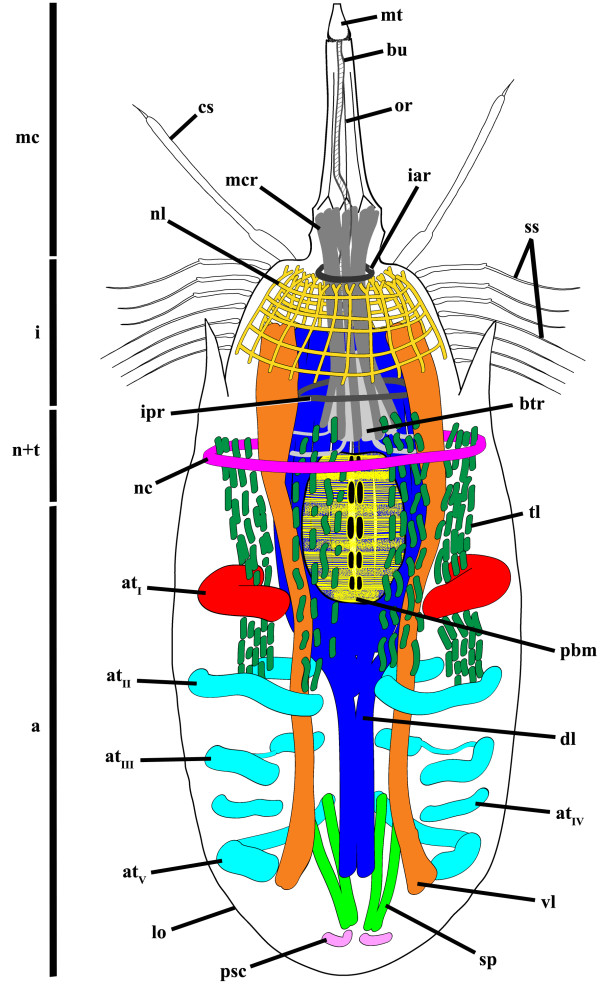
***Nanaloricus *sp. Schematic drawing of the myoanatomy of an adult with fully extended head.** The outer contour of the specimens is represented by the black line. Note that the shape and the position of the scalids is merely representative. Abbreviations: a, abdomen; at_I-V_, abdominal transverse muscles; btr, buccal tube retractors; bu, buccal tube; cs, clavoscalid; dl, dorsal longitudinal retractor muscle; i, introvert; iar, introvert anterior ring muscle; ipr, introvert posterior ring muscle; lo, lorica; mc, mouth cone; mcr, mouth cone retractors; mt, mouth tube; n + t, neck and thorax; nc, neck circular muscle; nl, net-like thin muscles; or, oral ridge; pbm, pharyngeal bulb musculature; psc, posteriormost short curved muscles; ss, spinoscalid; sp, short posterior longitudinal muscles; tl, thin longitudinal muscles; vl, ventral longitudinal retractor muscle.

The musculature of adult *Nanaloricus* sp. includes several paired arrays of muscle fibers arranged in longitudinal, transverse or circular orientations. A net-like muscular structure is found in the head region (Figures 1B-D, 3B, 4B-D, 5B,D, 6). This structure is composed of five thin circular fibers arranged in parallel around the introvert, which are crossed by several (up to 30) thin longitudinal fibers. These thin longitudinal fibers extend from the posteriormost to the anteriormost region of the net-like structure; the posterior tip of each of the longitudinal fibers is single, while its anterior extremity is double. The net-like arrangement of this structure is found in almost all specimens analyzed except in one where the thin circular fibers are located anteriorly and not intercrossed by the thin longitudinal fibers (Figure 2B-F).

**Figure 7:**
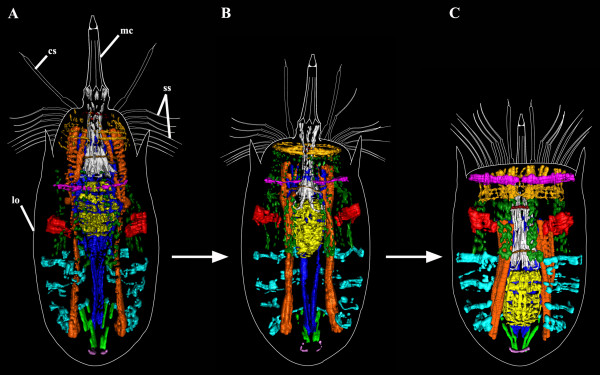
***Nanaloricus *sp. Comparative diagram of the 3D rendered muscular system at different stages of body retraction.** The outer contour of the specimens is represented by the white lines, while the arrows indicate the transition from fully extended to retracted body. Anterior faces upwards in all aspects. **A**: Specimen with fully extended introvert. The prominent pharyngeal bulb musculature is located anteriorly to the abdominal transverse muscle I. The dorsal and ventral retractors span the head region, which is characterized by the presence of a net-like muscular structure. **B**: Specimen with slightly retracted introvert. The pharyngeal bulb musculature is located between abdominal transverse muscle I and II. Note that the net-like thin muscles in the head are arranged as a thick, ring-like structure. **C**: Specimen with introvert retracted. The neck circular muscle merges the net-like thin muscles in the head. The pharyngeal bulb musculature is located posteriorly in the abdomen, between abdominal transverse muscle II and V. Color code: abdominal transverse muscle I, red; abdominal transverse muscles II-V, light blue; buccal tube retractors and mouth cone retractors, white; dorsal longitudinal retractor muscle, dark blue; introvert anterior ring muscle, dark red; introvert posterior ring muscle, brown; neck circular muscle, purple; net-like thin muscles, dark yellow; pharyngeal bulb musculature, yellow; short posterior longitudinal muscles, green; posteriormost short curved muscles, light purple; thin longitudinal muscles, dark green; ventral longitudinal retractor muscle, orange. Abbreviations: cs, clavoscalid; lo, lorica; mc, mouth cone; ss, spinoscalid.

The neck region is characterized by the presence of a thick, circular muscle composed of 2–3 fibers (Figures 1C-F, 2B-F, 3B, 4B-D, 5B,D, 6). The arrangement of this muscle in relation to the other sets of muscle varies when the animal retracts or extends the head (Figure 7A-C). However, the neck circular muscle is always located externally to all longitudinal muscles spanning along the neck region, and anteriorly to the first pair of transverse muscles. In specimens with extended head, the net-like myoanatomical structure is located anteriorly to the neck circular muscle (Figures 1B-E, 2B-E, 3B, 6, 7A). On the contrary, in specimens with fully retracted head the net-like structure is situated internally (Figures 2B-E, 7A) or posteriorly to the neck circular muscle (Figures 5B,D).

In the abdomen, five pairs of transverse muscles are arranged laterally and form the outermost body wall musculature (Figures 1B-E, 2B-E, 3B, 4B-D, 5B,D, 6). The first (anteriormost) transverse muscle, the thickest within this group, is composed of 6–8 fibers. This muscle has a semicircular shape, in which the open side faces the longitudinal muscles running internally in the abdominal cavity. The ventral and dorsal ends of the anteriormost transverse muscle inserts on the ventral and dorsal thin longitudinal muscles, respectively (Figures 1B-E, 2B-E, 3B, 4B-D, 6). In some specimens, the dorsal end also inserts on the dorsal longitudinal retractors (Figures 1D,E, 4D). The second to fifth (posteriormost) transverse muscles possess a semicircular arrangement as well, although they differ in composition and length (Figures 1B-E, 2B-E, 3B, 4B-D, 5B,D, 6). The second transverse muscles span from the posteriormost extremity of the ventral thin longitudinal muscles to the middorsal region of the body, very close to the dorsal longitudinal retractors. These muscles are not continuous because a large gap is present laterally dividing the ventral and dorsal parts (Figures 1C-E, 2C-E, 4C-D). The third transverse muscle is arranged like the second, however the gap is interrupted posteriorly by a very thin fiber that connects the ventral and dorsal parts (Figures 1C-E, 2C-E, 4C-D). Both the second and the third transverse muscles are composed of 3–4 fibers. The fourth transverse muscle, composed of only 2 fibers, is the shortest within this group of semicircular muscles and spans only ventrolaterally (Figures 1C-E, 2C-E, 4C-D). Finally, the fifth (posteriormost) transverse muscle extends from the ventrolateral body region to the middorsal region of the body, virtually connecting to the anterior end of the short posterior longitudinal muscles (Figures 1C-E, 2C-E, 4C-D). Like the second and third transverse muscles, the fifth is composed of 3–4 fibers.

Two dorsal longitudinal retractor muscles emerge from the medial, posteriormost region of the body and span the entire length of the abdomen (Figures 1B,D,E, 2B,D,E, 4B,D, 5B,D, 6). After the first half of the abdomen length, these muscles split in up to 6 other longitudinal fibers in a fan-like arrangement. These fibers run in parallel through the anteriormost region of the abdomen into the thoracic/neck region. On the opposite region of the abdomen, two thick ventral longitudinal retractors emerge from the lateral-posteriormost region of the abdominal cavity and run in parallel to each other along the anterior-posterior body axis (Figures 1B-D, 2B-D, 4B,C, 5B,D, 6). Both fibers extend anteriorly into the thoracic/neck region, and terminate at the same body length level as the dorsal longitudinal retractors. When the animal is fully retracted, both dorsal and ventral longitudinal retractors end close to the anteriormost semicircular muscle (Figures 4B-D, 5B,D, 7A-C).

Several sets of wide, thin longitudinal muscles are distributed radially in the head and thoracic/neck region and extend into the anteriormost region of the abdomen (Figures 1B-E, 2B-E, 3B, 4B-D, 5B,D, 6, 7A-C). Two ventral thin longitudinal muscles emerge externally to the anteriormost region of the ventral longitudinal retractors. A second set of these longitudinal muscles is located laterally and emerges internally to the two anteriormost transverse muscles. Finally, a third set of thin longitudinal muscles is located dorsally and anteriorly to the dorsal longitudinal retractors. In fully retracted specimens, the thin longitudinal muscles fold inwards as the head is completely drawn back inside the abdominal cavity (Figures 5B,D).

Two short longitudinal muscles, situated in the dorsal-posteriormost region of the abdominal cavity, are composed of two pairs of fibers arranged approximately in a V-shaped orientation (Figures 1B-E, 2B-E, 3B, 4B-D, 5B,D, 6). The two muscles merge toward the midline of the body and insert posteriorly. An additional pair of short curved muscles is present in the ventral-posteriormost region of the abdomen, close to the anal pore (Figures 1B-E, 2B-E, 3B, 4B-D, 5B,D, 6). In some specimens these short curved muscles are surrounded anteriorly and laterally by a small number of very thin fibers, which are probably part of the visceral musculature (Figures 2B, 4B).

The pre-pharyngeal armature, situated anteriorly to the pharyngeal bulb, is surrounded by two sets of muscles located very close to each other (Figures 2A, 4A, 5B, 6). The inner set is composed by 6 buccal tube retractors with a broad base, which are arranged 3 × 2 and diagonally in a conical shaped structure (Figures 1B,C, 2B,C, 3B, 4B,C, 5B, 6). Additionally, a total of 6 thin fibers connect the base of this conical structure to the ventral and dorsal longitudinal retractors (Figures 1C, 2C, 4C, 6). The outer set of muscles is constituted by 8 mouth cone longitudinal retractors, which are arranged around the conical muscle structure. The mouth cone retractors run anteriorly, along the buccal tube, until the base of the mouth cone region where they appear to attach (Figures 1B-F, 2BF, 3B, 4B-D, 5B,D, 6). Two ring muscles are located externally to these two sets of muscles: the introvert posterior ring is located midway the length of the conical shaped structure (Figures 1C,E,F, 2B,C, 3B, 4B,C, 6), while the introvert anterior ring is located halfway along the length of the mouth cone retractors (Figures 1C-F, 2C-F, 4B-D, 6). The anterior ring is smaller in diameter than the posterior ring muscle.

The large, oval-shaped pharyngeal bulb is composed of at least six fibers arranged transversally, of which the posteriormost is the thinnest fiber (Figures 1B,C, 2B,C, 3B, 4B,C, 6). The position of this structure within the abdominal cavity varies, depending on the condition of the specimen analyzed: in animals with fully extended introvert the pharyngeal bulb is located anteriorly to the first pair of transverse muscles (Figures 1C, 7A), while in retracted animals this myoepithelial organ is situated posteriorly to the second pair of transverse muscles (Figures 4C, 7C).

### Gross morphology of the *Armorloricus elegans* Higgins larva

**Figure 8:**
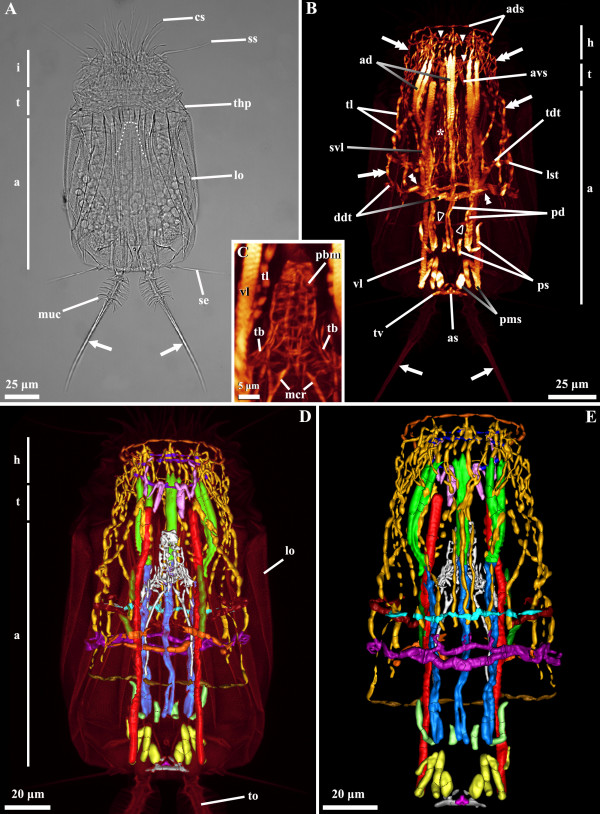
***Armorloricus elegans.*** Light micrography of a Higgins larva stage (**A**). Body musculature visualized by fluorescently-labeled phalloidin staining and CLSM (**B** and **C**) and by 3D imaging (**D** and **E**). Anterior faces upwards in all aspects. **A**: Dorsal view. Specimen with fully retracted mouth cone (indicated by the dashed line). Note the posterior toes with cut-off mucrones. **B**: Ventral view. The myoanatomy is more complex in the anterior half of the body. Note that the posterior toes lack muscles inside. **C**: Close up of the mouth cone musculature. Dorsal view. **D**: Dorsal view. **E**: Ventral view. Color code: anal sphincter, pink; anterior ring muscle, dark blue; anterodorsal longitudinal retractor muscles, green; anterodorsal semicircular muscle, dark orange; anteroventral short longitudinal muscles, light purple; dorsal double transverse muscle, purple; lateral short transverse muscle, dark red; mouth cone musculature (including pharyngeal bulb and mouth cone longitudinal muscles), white; posterior short longitudinal muscles, light green; posteriormost short longitudinal muscles, yellow; posterodorsal longitudinal retractor muscles, blue; short midventral longitudinal muscles, dark green; thin longitudinal muscles, dark yellow; thin middorsal transverse muscle, light blue; thin posteroventral transverse muscle, brown; triangular posteroventral muscle, grey; ventral double transverse muscle, orange; ventral longitudinal retractor muscles, red. Abbreviations: *, mouth cone musculature; a, abdomen; ad, anterodorsal longitudinal retractor muscles; ads, anterodorsal semicircular muscle; avs, anteroventral short longitudinal muscles; as, anal sphincter; cs, clavoscalid; ddt, dorsal double transverse muscle; i, introvert; lo, lorica; lst, lateral short transverse muscle; mcr, mouth cone longitudinal retractors; muc, mucrones; pbm, pharyngeal bulb musculature; pms, posteriormost short longitudinal muscles; ps, posterior short longitudinal muscles; pd, posterodorsal longitudinal retractor muscles; se, posterolateral sensory setae; ss, spinoscalids; svl, short midventral longitudinal muscles; t, thorax; tb, thin longitudinal muscles of the base of the mouth cone; tdt, thin middorsal transverse muscle; thp, thoracic plates; tl, thin longitudinal muscles; tv, triangular posteroventral muscle; vl, ventral longitudinal retractor muscles; arrows, posterior toes; arrowheads, anterior ring muscle; double arrows, thin longitudinal muscles; double arrowheads, ventral double transverse muscle; open arrowheads, thin posteroventral transverse muscle.

The external morphology of the larval stages investigated in this study is in accordance with the description of the Higgins larva that Kristensen & Gad [2] refer to as *Armorloricus* sp. I. Its body is divided into four main regions: mouth cone, introvert, thorax, and abdomen. The mouth cone is conical and flexible. Six rows of scalids cover the introvert, which is spherical in shape. The thoracic region is flexible and accordion-like. The loricated, rectangular abdomen possesses a sculptured cuticle and three well-developed papillate flosculi located dorsal-posteriorly (Figure 8A; [2]). Ventral locomotory setae and sensory setae are situated at the anteriormost and the posterior region of the lorica, respectively. In addition, the typical posterior toes possess short, cut-off mucrones with smooth rims. Indeed, this morphology of the toes is found only in Higgins larvae associated with adults of *Armorloricus elegans*, which enables the assignment of *Armorloricus* sp. I to this species (RMK, pers. obs.).

### The myoanatomy of the *Armorloricus elegans* Higgins larva

**Figure 9:**
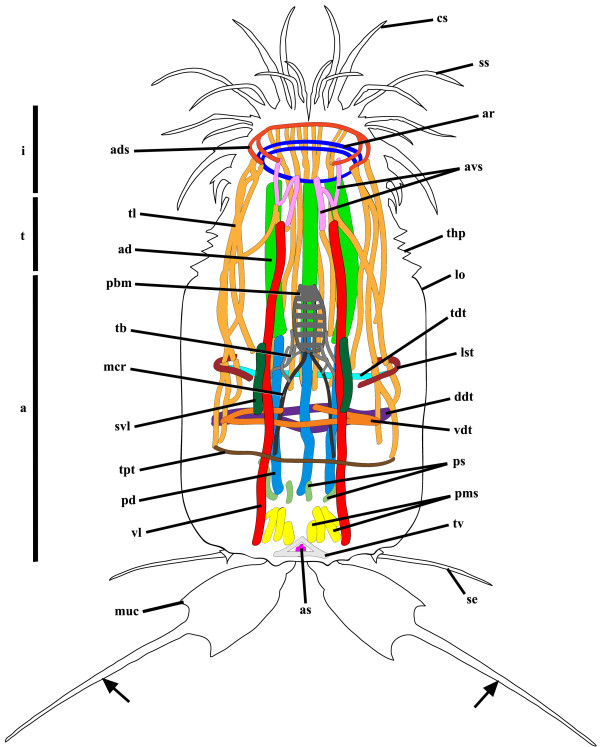
***Armorloricus elegans*.** Schematic drawing of the myoanatomy of a larva with retracted mouth cone. The outer contour of the specimen is represented by the black line. Abbreviations: a, abdomen; ad, anterodorsal longitudinal retractor muscles; ads, anterodorsal semicircular muscle; ar, anterior ring muscle; avs, anteroventral short longitudinal muscles; as, anal sphincter; cs, clavoscalid; ddt, dorsal double transverse muscle; i, introvert; lo, lorica; lst, lateral short transverse muscle; mcr, mouth cone longitudinal retractors; muc, mucrones; pbm, pharyngeal bulb musculature; pd, posterodorsal longitudinal retractor muscles; pms, posteriormost short longitudinal muscles; ps, posterior short longitudinal muscles; se, posterolateral sensory setae; ss, spinoscalids; svl, short midventral longitudinal muscles; t, thorax; tb, thin longitudinal muscles of the base of the mouth cone; tdt, thin middorsal transverse muscle; thp, thoracic plates; tl, thin longitudinal muscles; tpt, thin posteroventral transverse muscle; tv, triangular posteroventral muscle; vdt, ventral double transverse muscle; vl, ventral longitudinal retractor muscles; arrows, posterior toes.

The body musculature of the Higgins larvae of *Armorloricus elegans* includes several pairs of longitudinal fibers that are present on both sides of the larval body (Figure 8B-E, 9). A pair of ventral longitudinal retractors, present around the midline, extends from the posteriormost region of the abdomen into the thoracic region (Figures 8B,D,E, 9). An additional pair of midventral longitudinal muscles is present adjacent to the ventral longitudinal retractors. This pair is composed of short ventral longitudinal muscles, which run solely in the midway of the larval body length (Figures 8B,D, 9).

Dorsally, two groups of three longitudinal retractors extend contiguously along the body length (Figures 8B,E, 9). The anterodorsal longitudinal retractors extend from the head region to the first third of the abdominal cavity, while the posterodorsal longitudinal retractors extend from the upper-third of the abdomen into the posterior third of the abdomen. The anterodorsal longitudinal retractors are thicker than the posterodorsal longitudinal retractors, and all muscles appear to be composed of two fibers that extend parallel to each other.

The first half of the whole larval body is characterized by the presence of several thin longitudinal fibers that extend from the anteriormost region of the head into the first half of the abdomen (Figures 8B,D,E, 9). These thin longitudinal muscles run both on the ventrolateral and the dorsal side of the larval body and intercross several transverse muscles situated in the second third of the abdomen length. Ventrally, a thin posterior transverse muscle extends from the right side to the left side of the larval body, and its extremities connect to the terminations of the thin longitudinal muscles. Another ventral transverse muscle runs more anteriorly and is composed by two fibers arranged parallel to each other but converging laterally. This transverse muscle is arranged externally to the ventral longitudinal retractors, and extends from the right side to the left side of the body (Figures 8B,D, 9).

Dorsally, two transverse muscles extend from the left to the right side of the larval body. A double transverse muscle is arranged externally to the posterodorsal longitudinal retractors, in the region opposite to the ventral double transverse muscle. The dorsal double transverse muscle is composed of two thick fibers; the anteriormost fiber connects to the posterodorsal longitudinal retractors via short, thin fibers (Figures 8B,E, 9). Anteriorly to this muscle, a thin dorsal transverse muscle crosses the posterodorsal longitudinal retractors and at least eight thin longitudinal muscles externally (Figures 8B,E, 9). Each extremity of this muscle connects to a lateral short transverse muscle (Figures 8B,D,E). These lateral muscles are curved in shape and connect to thin longitudinal muscles both on the ventrolateral and dorsolateral region of the body.

Circular muscles characterize the musculature of the introvert (Figures 8B,D,E, 9). The anteriormost of these muscles is a semicircular structure, which is composed of a single fiber running dorsally and possesses ventrolateral extremities with two fibers (Figures 8D,E, 9). The introvert musculature also includes a thin ring muscle, which is composed of two fibers on the dorsal side and a single fiber on the ventral side (Figures 8D,E, 9). Two pairs of short, anteroventral longitudinal muscles cross the introvert ring muscle on the ventral side (Figures 8B,D, 9). The innermost pair, which is the thickest and shortest, emerges close to the ventral longitudinal retractors. The outermost pair of the anteroventral muscles crosses the introvert ring, as well as the extremities of the anteriormost semicircular muscle. Additionally, several thin longitudinal muscles cross or connect to the introvert circular muscles, both dorsally and ventrolaterally (Figures 8B,D,E, 9).

In the rear part of the abdomen, two groups of short longitudinal muscles are found. In one group, four posterior short muscles are arranged alternating with the posterodorsal longitudinal retractors (Figures 8B,D, 9). The other group includes the four posteriormost short muscles, which are arranged in between the ventral longitudinal retractors (Figures 8B,E, 9). In the latter group the innermost pair has a round shape, while the two outermost pairs connect to the ventral longitudinal retractor. In addition, the posteriormost muscles in the larval body include a ventral triangular muscle and a small anal sphincter located dorsally (Figures 8B,D,E, 9).

Finally, the musculature of the mouth cone is composed by the pharyngeal bulb muscles and a number of longitudinal muscles. In the pharyngeal bulb, four thin fibers run longitudinally ― two medially and two laterally―, and up to seven thick fibers run transversally (Figures 8B,C, 9). The anteriormost fiber appears very thick in comparison to the other transversal fibers. Several thin longitudinal fibers (4–6) are found at the base of the mouth cone, connecting to the pharyngeal bulb (Figures 8C, 9). Additionally, a pair of longitudinal retractors emerges from the base of the mouth cone and, when this structure is retracted, it spans into the second half of the abdomen length (Figures 8C,D,E, 9).

## Discussion

### Comparative myoanatomy of the adult stage of *Nanaloricus* sp

Prior to this study, *Nanaloricus mysticus* was the only species within family Nanaloricidae studied at the myoanatomical level, though a few transmission electron micrographs of the head region of *Armorloricus elegans* are available in the literature (cf. [12,20,25]; see Table 1). The musculature of *Nanaloricus* sp. described here differs somewhat from that of *N. mysticus* revealed by ultrastructural examination. For instance, the radial muscles attached to the furcal base of the mouth cone of *N. mysticus* (see Figure six in [20]) were not found during this study in *Nanaloricus* sp. Otherwise, the 8 mouth cone retractors found in *Nanaloricus* sp. seem to correspond to the 8 homonymous muscles found inside the ridges of the mouth cone of *N. mysticus*, as well as in *Pliciloricus enigmaticus*, *P. pedicularis*, *P. diva* and *Rugiloricus doliolius*[10,11,16,20].

In the head, *N. mysticus* possesses 5 inner and 15–24 outer longitudinal muscles (named head retractors) and 2–3 circular muscles surrounding the brain, which is a condition found also in *P. enigmaticus*[20]. These observations are hard to compare with the description achieved here for *Nanaloricus* sp., because in extended specimens the head region accommodates several longitudinal muscles, e.g., the thin longitudinal muscles, the dorsal and ventral retractors, and the mouth cone retractors. However, based on the line drawings of *N. mysticus*, from Kristensen ([20], see Figure seven therein) and Bang-Berthelsen et al. ([12], see Figure 6.1.1), we speculate that the inner and outer longitudinal head retractors may be compared, respectively, to the posterior part of the mouth cone retractors (located externally to the buccal tube retractors) and the anterior extremity of dorsal and ventral retractors observed in *Nanaloricus* sp.

The 2–3 inner circular muscles and several scalid ring circular muscles described in the head of *N. mysticus* (see Figure seven in [20]), as well as the 10 head circular muscles described in Bang-Berthelsen et al. [12], probably correspond to the net-like muscular arrangement described for *Nanaloricus* sp. in this study. However, it is not clear whether or not the arrangement of the 4 thin circular fibers and radially arranged short fibers of the net-like muscular structure correspond to the position of any of the rings of scalids in the head. In case the net-like structure is associated with the scalids, it is interesting to note that *P. enigmaticus* possesses only 5 head circular muscles associated to the rings of scalids, which would resemble more the condition found in *Nanaloricus* sp. [20].

The occurrence of muscles inside the spinoscalids of *N. mysticus*, *A. elegans*, *P. enigmaticus*, *P. diva*, *R. doliolius* and *R. renaudae* was described in earlier studies [11,13,16,20,25]. Ultrastructural analysis in *N. mysticus* and *A. elegans* revealed a pair of small muscles in the spinoscalids of the anteriormost rows and the same condition was described for *P. enigmaticus*, except for the two midventral enlarged scalids of the second ring where up to five muscles are found [12,20,25]. In our study, similar muscles were not clearly observed inside the clavoscalids or the spinoscalids of *Nanaloricus* sp., and the net-like structure is the myoanatomical feature located closest to these locomotory/sensory appendages. Therefore, it cannot be excluded that the anterior double extremities of the thin longitudinal fibers composing the net-like structure are related to the clavoscalids, while the five thin circular fibers and the posterior single extremities of these thin longitudinal fibers are related to the following rows of spinoscalids, i.e., the second to the seventh row of head appendages. The absence of muscles related to rows eight and nine is probably due to the fact that in family Nanaloricidae these rows are composed of trichoscalid-like and beak-like appendages, respectively, which are merely sensory structures. In the future, ultrastructural analyzes on *Nanaloricus* sp. will be important to clarify whether or not the net-like structure is associated with the scalids and if these head appendages possess muscles internally.

The buccal tube retractors described in *N. mysticus* possess a 3 × 2 organization and are located surrounding the posterior region of the buccal tube, which connects to the hexagonal pre-pharyngeal apparatus (see Figures seven and eight in [20]). Likewise, the conical shaped muscle structure found surrounding the pre-pharyngeal apparatus of *Nanaloricus* sp. is composed of 3 × 2 buccal tube retractors arranged diagonally. Based on these similarities, it is suggested that the 3 × 2 arranged buccal tube retractors is a trait shared by these two nanaloricid species. Moreover, this might be a trait found only in family Nanaloricidae, because in *P. enigmaticus* the pre-pharyngeal apparatus is absent and the relatively small pharyngeal bulb is enclosed in the mouth cone [20].

A number of circular muscles occur in the neck of *N. mysticus*[12,20]. In fully extended specimens these muscles appear to surround the pharyngeal bulb and longitudinal retractors, which is similar to the condition found in the species studied here. However, in our analysis on *Nanaloricus* sp. we found only one neck circular muscle. In *P. enigmaticus* it is known that circular muscles occur not only in the neck but along the entire body length, although information on how many of these muscles are present in the neck is not available [12].

Within the abdomen, longitudinal and circular (or transverse) muscles are found in *N. mysticus*, *P. enigmaticus*, *P. pedicularis*, *P. diva*, *R. doliolius* and *R. renaudae*[10,11,13,16,20]. The dorsal longitudinal retractor muscle found in *Nanaloricus* sp. clearly resembles the dorsal muscle described for *N. mysticus* (see Figures ten and eleven of [20]) or for *P. enigmaticus* (see Figures twenty to twenty two of [20]). Interestingly, the dorsal muscle of *N. mysticus* splits in two in its posteriormost section (see Figures 10 and 11 of [20]). Since a pair of short posterior longitudinal muscles is present in *Nanaloricus* sp., it cannot be excluded that the double terminal end of the dorsal muscle of *N. mysticus* is instead a pair of short posterior muscles; more studies are necessary to confirm this. In addition, the pair of lateral longitudinal muscles located in the trunk of *P. enigmaticus* (see Figures twenty to twenty two of [12,20]) has an arrangement similar to the posterior region of the ventral longitudinal retractor muscles described here for *Nanaloricus* sp. Other longitudinal muscles are described in the abdomen of *P. enigmaticus*, as well as in *P. pedicularis*, *P. diva*, *R. doliolius* and *R. renaudae*, though more analyses are necessary to better understand their arrangement [10,11,13,16,20].

The abdomen of *N. mysticus* is characterized by the presence of several dorsal-ventral muscles, as well, though the exact arrangement and number of these muscles is not clear [20]. However, it appears that a large dorso-ventral muscle is located anteriorly and ca. 7 thin dorso-ventral muscles are arranged along the abdominal length of *N. mysticus*, which is similar to the arrangement of the 5 pairs of transverse muscles of *Nanaloricus* sp. Contrary to this condition, in family Pliciloricidae the circular muscles of the abdomen appear to be present in high number. For instance, *P. enigmaticus* possesses 11 circular muscles in the abdominal cavity, which are described as ring-like [20], while in other pliciloricid species, e.g., *P. pedicularis*, *R. doliolius* and *R. renaudae*, circular muscles of the abdomen are described as many bundles [10,13,16]. The actual number of these bundles is not reported.

Earlier observations on the ultrastructure of *N. mysticus* and *P. enigmaticus* revealed a radial arrangement of the transversal fibers composing the pharyngeal bulb [12,20]. Likewise, our results show that the pharyngeal bulb of adult *Nanaloricus* sp. is composed of ca. 6 transversal fibers, however, observations at the ultrastructural level are necessary to confirm their radial arrangement. Moreover, knowledge about the number of radial muscles is lacking both in *N. mysticus* and in *P. enigmaticus*, though light microscopy observations show that only 3 layers of these muscles are present in the pharyngeal bulb of another loriciferan, *P. pedicularis*[10]. Therefore, the number of transversal muscles in the pharynx bulb of *Nanaloricus* sp., a representative of family Nanaloricidae, is twice as much as that found in *P. pedicularis*, a representative of family Pliciloricidae. Since in adults of family Pliciloricidae the pharyngeal bulb is located inside the mouth cone, the reduced number of transversal muscles composing this myoepithelial structure might be related to anatomical constraints, i.e., the restricted volume available internally.

Generally, the description of various myoanatomical features found in the distinct body regions of the adult *Nanaloricus* sp. confirms earlier observations on adult stages of other loriciferan species. However, various muscles are described here for the first time in adult loriciferans. This is the case for the two ring muscles found in the introvert, located externally to the buccal tube and mouth cone retractors, and for the pair of short, curved muscles located in the ventral-posteriormost region of the abdomen. These muscles are all very thin and occupy a very small volume within the adult body, which makes the analysis by light or transmission electron microscopy very difficult. Phalloidin labeling of F-actin present in muscles combined with CLSM and 3D reconstruction is thus an ideal complement for investigations of the myoanatomy of Loricifera in detail.

### Functional implications of the myoanatomy of the *Nanaloricus* sp. adult stage

The motion of the adult stages of family Nanaloricidae is characterized by short backward movements followed by forward jumps, which is accompanied by the retraction and extension of the head, respectively (Additional files 1 and 2; [12]). Therefore, all muscles involved in the extension/retraction of the head most probably contribute to this behavior. Accordingly, the dorsal and ventral longitudinal retractors are putatively responsible for the retraction, while the various thin longitudinal muscles appear to play an antagonistic role leading to the extension of the head. This might be the reason why the thin longitudinal muscles fold inwards in fully retracted specimens, whereas in specimens with the head out these thin muscles are completely extended. Moreover, the clavoscalids and the anteriormost rows of spinoscalids appear to be used as propulsive structures during the forward jumps. Since it is not clear whether or not the adult *Nanaloricus* sp. possesses muscle fibers inside the head external appendages, the net-like muscular structure is interpreted here as responsible for the movement of the clavoscalids and the anteriormost rows of spinoscalids. Consequently, the contraction of the circular and longitudinal muscles composing the net-like structure would lead to the retraction of the head appendages, which are accommodated inside the abdominal cavity in fully retracted specimens. Additionally, the double anterior extremity of the thin longitudinal muscles composing the net-like structure might indicate the presence of an antagonistic pair of muscles participating in the movement of the clavoscalids.

Further, observations on the jumping behavior of adult nanaloricids revealed that the mouth cone is protruded synchronously with the extension of the head and the forward jumps. In *Nanaloricus mysticus*, 8 mouth cone retractors and 6 buccal tube retractors are responsible for the telescoping of the mouth cone and the extrusion of the buccal tube, respectively [21]. Likewise, the mouth cone and buccal tube retractors found in *Nanaloricus* sp. most probably have a similar role during the typical jumping behavior. In addition, the telescoping of the mouth cone, as well as the upward-downward movements of the pharyngeal bulb, might be assisted to some extent by the ventral and dorsal longitudinal muscles. This is because of (i) the presence of 6 thin fibers connecting the buccal tube retractors (i.e., the conical muscle structure surrounding the pre-pharyngeal armature) to the dorsal and ventral retractors, (ii) the close proximity between the conical structure and the mouth cone retractors, and (iii) the absence of retractors anchored to the pharyngeal bulb. Accordingly, the contraction of the dorsal and ventral retractors is likely to cause the conical structure and the mouth cone retractors to move downwards, which in its turn drags the mouth cone and the pharyngeal bulb posteriorly.

The transverse muscles present in adult *Nanaloricus* sp. appear to give the body structural support (by holding the lorica plates together) and mechanical protection, especially when the animal retracts the head and mouth cone inside the abdominal cavity. However, an auxiliary role of these laterally arranged muscles while the animal moves cannot be ruled out. Contraction of the transverse muscles likely increases the internal pressure within the abdominal cavity, which might facilitate the projection of the head out of the abdominal cavity. The neck circular muscle might have a mechanical defensive role as well. This muscle is probably a sphincter-like muscle that is responsible for the occlusion of the anteriormost region of the abdominal cavity while the head is retracted. The protection offered by this muscle to the head region appears thus to be a complement to the anteriorly oriented spikes of the lorica. Furthermore, due to its arrangement the neck circular muscle might be related to the movement of the trichoscalids.

The function of the various small muscles found along the anterior-posterior body axis is not clear. This is the case for the two introvert ring muscles located externally to the buccal tube retractors and mouth cone retractors. Since both ring muscles are far from the mouth opening, their role as sphincter muscles is unlikely. Therefore, the anterior ring muscle is probably a structural muscle subsidiary to the mouth cone retractors, while the posterior ring muscle possibly supports the action of the buccal tube retractors and the dorsal and ventral retractors. The pair of short curved muscles present in the ventral-posteriormost region of the abdomen is interpreted as an anal sphincter due to its location. However, this assumption must be validated by observations at the ultrastructural level.

### Comparative myoanatomy of the *Armorloricus elegans* Higgins larva stage

Comprehensive information concerning the muscle architecture of loriciferan larval stages is scarce. Prior to this study, morphological data on the myoanatomy of the Higgins larva stage was available only from species belonging to family Pliciloricidae. Accordingly, light microscopy observations revealed the presence of 6 short mouth cone retractors, 8 introvert retractors and 8 trunk retractors in *Rugiloricus doliolius*[16], and of an unspecified number of trunk retractors in *Pliciloricus pedicularis*[10]. The number of muscles found is probably underestimated due to the limitations of the technique used to study myoanatomical features, though it is clear that longitudinal muscles are present in the distinct body regions of the Higgins larva of Pliciloricidae. In addition, 7 ring muscles are present in the abdominal cavity of *R. doliolius*[16], while in *P. pedicularis* an unspecified number of abdominal transverse muscles are found. Since the longitudinal retractors of the mouth cone, introvert, and abdomen, and abdominal transverse muscles are found in *Armorloricus elegans* as well, these muscle subsets can be assigned to the myoanatomical ground pattern of the Higgins larva.

Earlier studies pointed out the presence of small muscles inside the scalids and the bases of the toes in the Higgins larva of *Rugiloricus doliolius*[16]. However, the results obtained during our study do not provide evidence for any muscle fibers inside these structures. In the Higgins larva of *A. elegans*, the anterodorsal semicircular muscle and the anteriormost thin longitudinal muscles are the myoanatomical subsets located closest to the spinoscalids, in the introvert. As for the posterior toes, the triangular posteroventral muscle and the posteriormost short longitudinal muscles are the only myoanatomical structures found in the vicinity of this locomotor organ. In the future, new observations based on electron microscopy are necessary to clarify the presence of these muscle sets in the Higgins larva of *A. elegans*. In addition, two narrow ring muscles are present in each row of thoracic plates in the Higgins larva of *R. doliolius*[16]. This condition is different from that found in the Higgins larva of *A. elegans*, which possesses a single ring muscle located in the introvert and none in the thorax. Such a difference is thus an important character to distinguish between family Nanaloricidae and Pliciloricidae.

The pharyngeal bulb of *P. pedicularis* and *Pliciloricus diva* is described as possessing 5 layers of 3 radial muscles each [10,11]. The condition found in *A. elegans* is different mainly because of the presence of four thin longitudinal fibers, which are not described in these two pliciloricid species. In addition, the pharyngeal bulb of *A. elegans* is composed of more transversal (radial) layers than that of *P. pedicularis* and *P. diva*. This myoepithelial structure seems thus to be more complex in Nanaloricidae than in Pliciloricidae, however more species from both families must be analyzed in better detail before any final conclusion.

### Functional implications of the myoanatomy of the *Armorloricus elegans* Higgins larva stage

In family Nanaloricidae, the Higgins larva stage uses the posterior pair of toes to perform slow, walking-like movements (pers. obs.). Since muscle fibers were not found inside the toes of *Armorloricus elegans*, the movement of these locomotory structures is probably controlled by the triangular posteroventral muscle and the inner pairs of the posteriormost short longitudinal muscles, because of their arrangement in close proximity to the posterior toes. Further, the three pairs of ventral setae situated in the anteriormost region of the lorica are believed to be used as locomotory (or grasping) organs as well [2,21]. In this case, the pair of longitudinal short muscles located anteroventrally is probably responsible for operating the movement of these setae.

The two mouth cone retractors are responsible for the withdrawal of this structure into the abdominal cavity. This process is independent of the retraction of the introvert, which is probably carried out by the several thin longitudinal muscles and the 3 anterodorsal longitudinal retractors. Moreover, the circular anterior muscles present in the introvert may act as sphincter muscles to close the anteriormost region of the abdominal cavity while the whole head is retracted. The 3 posterodorsal and the ventral pair of longitudinal retractors may contribute to the retraction of the thoracic region of the larval body. The transverse muscles present in the body of the Higgins larva of *A. elegans* appear to have a mechanical protective role. In addition, these muscles may be important for increasing the internal pressure within the abdominal cavity in order to pull out the introvert or the mouth cone out of the abdominal cavity. Finally, the anal sphincter is the only sphincter-like muscle found along the through gut of the Higgins larva of *A. elegans*.

### Comparison with the myoanatomy of Kinorhyncha, Priapulida and Nematomorpha

The evolutionary relationships of loriciferans within Metazoa remain unclear ever since their discovery, although Phylum Kinorhyncha was suggested to be the sister group in the original description based on the gross morphology of loriciferans [1]. However, several potential morphological synapomorphies for Loricifera and Priapulida have also been suggested [1,29], and morphological as well as molecular evidence points towards a closer relationship with Nematomorpha [1,28]. A comparison of the musculature in the two examined species of Loricifera and their three potential sister groups would of course be interesting, though it should be performed with the utmost care. Indeed, even though loriciferans share common traits with all the three groups, they still differ fundamentally in general morphology, with the priapulids being thick and voluminous worms, kinorhynchs having a segmented trunk, and nematomorphs being extremely long and thin endoparasites.

Musculature of Priapulida has been examined at the ultrastructural level with transmission electron microscopy in both macroscopic [30-34] and microscopic species [35]. Recently, Rothe et al. [36] also studied the musculature of the microscopic *Tubiluchus troglodytes* using phalloidin staining and CLSM. It appears to be a common trait for both micro- and macroscopic priapulids to possess trunk muscles arranged in a densely packed grid of circular and longitudinal fibers (see illustrations in [36]). Even though both circular and longitudinal muscles are present in the examined species of *Nanaloricus* sp., the muscles are not in a grid-like arrangement, and we do not see any clear similarities between loriciferan and priapulid trunk musculature. Several muscles operate the priapulid introvert and mouth cone. From the larva of *Priapulus caudatus*, Lemburg [34] describes the presence of six large pharynx protractors that attach to the anterior part of the mouth cone (see Figure forty nine in [34]). In *Nanaloricus* sp. the mouth cone is extended into a long mouth tube, but in the anterior end of the mouth cone, at the point where the mouth tube extends from, we find eight mouth cone retractors. The anterior attachment of these muscles could suggest a homology with the pharynx protractors in *P. caudatus*, but the number of muscles (eight in Loricifera opposed to six in Priapulida) makes a homology uncertain. Pharynx protractors are also reported from *T. troglodytes* but the exact number of muscles is not clearly stated [36]. The retraction of the introvert in *P. caudatus* is operated by nine short introvert retractors that attach anteriorly near the anteriormost scalid ring, and posteriorly to the longitudinal trunk retractors. This composition contrasts to the situation in *Nanaloricus* sp. where it is the dorsal and ventral longitudinal trunk retractors that insert directly into the introvert. Circular muscles around the introvert are found in both *Nanaloricus* sp. and *T. troglodytes*, but whereas *Nanaloricus* sp. only has a few, clearly separated circular muscles, the circular muscles of *T. troglodytes* are numerous and part of a grid-like structure (formed together with the longitudinal muscles) that also characterizes the trunk region [36].

Kinorhynch muscular arrangement is best known from *Antygomonas* sp. and *Pycnophyes kielensis*, which both were examined by CLSM observations of phalloidin labeled specimens [37,38]. Due to the segmented nature of kinorhynchs and the plate composition of each segment, it is not useful to compare their trunk musculature with that of loriciferans. In contrast, the loriciferan and kinorhynch heads are quite similar, and this enables a more straight forward comparison. The study of *Antygomonas* sp. (see [37]) offers the best visualization of the head musculature. The mouth cone musculature includes fine muscles that innervate the nine outer oral styles. Deeper inside the mouth cone, sixteen mouth cone muscles are present. These mouth cone muscles extend from the pharynx, and anteriorly they nearly fuse pair-wise so that only eight muscles appear in the anterior part of the mouth cone. In addition, eight short basal mouth cone muscles overlap slightly with the sixteen mouth cone muscles and insert between the posterior extremities of each pair. Further, ten longitudinal pharynx protractors attach at the base of the mouth cone forming a muscular sheath together with the more posterior inner retractors. Circular muscles include two broad, slightly conical rings that wrap the introvert, and two more posterior ones that are associated with the neck region and hence are likely to be involved in the closing of the head opening.

The eight mouth cone retractors found in *Nanaloricus* sp. could be homologous with the sixteen mouth cone muscles found in *Antygomonas* sp. In both species the muscles extend from a pre-pharyngeal armature and anterior into the mouth cone, and even though the number of muscles is doubled in *Antygomonas* sp., the anterior, pair-wise fusion of the muscles indicates that the number of muscles can change quite easily from sixteen to eight. Pharynx protractors, as demonstrated in *Antygomonas* sp., could not be identified in *Nanaloricus* sp.

Nematomorphs possess a biphasic life cycle with an endoparasitic larva and short-lived, partly free-living adults. The adults are very long, thin worms, often without a mouth and with strongly reduced digestive system, and there is nothing in their gross morphology that would be comparable with loriciferans. The larvae though, are minute and equipped with a stylet and an introvert, which enables a much more meaningful comparison with loriciferans. The only CLSM study of larval nematomorphs was done on *Gordius aquaticus*[39]. Its trunk musculature consists mainly of longitudinal muscles, arranged in four bundles, and there are no obvious similarities with the muscular arrangement in loriciferans. However, some interesting traits can be observed in the musculature of the head. This includes three sets of longitudinal muscles that all have their posterior attachment point at the septum: six proboscis muscles extend from the distal end of the stylet, six oblique muscles extend from the position of the anteriormost hook ring, and twelve anterior parietal muscles extend from the septum to the posterior region of the introvert. The position of the six oblique muscles compares well to the position of the eight mouth cone retractors in *Nanaloricus* sp., but the number of muscles obviously differs between the two groups. We did not find any muscles extending into the mouth stylet in *Nanaloricus* sp., but interestingly the six buccal tube retractors in *Nanaloricus mysticus* extend their cuticular attachment fibers throughout the entire length of the buccal tube and attach near its distal tip. This arrangement is quite similar to the proboscis muscles in the *Gordius* larva.

## Conclusions

Our study provides new insight into the body musculature of the adult and Higgins larva stage of Loricifera. Indeed, several myoanatomical features revealed here for the first time contribute to a better understanding of the loriciferan anatomy. Based on the currently available knowledge, the myoanatomical bodyplan of the loriciferan adult stage includes: (i) 8 mouth cone retractors, (ii) a pharynx bulb composed of transversal fibers arranged radially, (iii) circular muscles present in the head and neck, (iv) internal muscles of the spinoscalids, (v) longitudinal muscles spanning the entire body, and (vi) transverse (circular) muscles in the abdominal cavity. In addition, other traits may contribute to the adult myoanatomical bodyplan. These are, e.g., the internal muscles of the clavo- and spinoscalids, and the net-like structure of the introvert, though the presence and arrangement of both features need to be studied thoroughly in all families of Loricifera. Concerning the Higgins larva, the muscle subsets assigned to its myoanatomical ground pattern are the (i) longitudinal retractors of the mouth cone, introvert, and abdomen, (ii) abdominal transverse muscles, and (iii) a pharynx bulb composed of transversal, radial fibers. In addition, the circular muscles of the introvert and the posterior triangular muscle in the abdomen discovered during this study are most probably part of the myoanatomical ground pattern of the Higgins larva, though more studies are needed to confirm this assumption.

Comparison of musculature in loriciferans and their three potential sister-taxa, i.e., Kinorhyncha, Priapulida and Nematomorpha, does not point unambiguously towards one specific closest relative. However, loriciferan musculature seems to have less in common with the patterns observed in priapulids, compared to the two other groups, kinorhynchs and nematomorphs. What is clearly supported by our data though is that similar traits can be found in the head musculature of all three scalidophoran taxa, as well as in the nematomorphs, which support the homology of introvert and head morphology across the cycloneuralian taxa.

## Materials and methods

### Collection of specimens and phalloidin staining

Specimens of adult *Nanaloricus* sp. and larval *Armorloricus elegans* were obtained from very clean, coarse shell gravel collected off the coast of Roscoff, France, in a location known as Trezen ar Skoden (see, e.g., [2]). The samples were taken with a Sanders dredge, at about 50 m depth, from four different transects: (i) 48°45’514”N/04°05’610”W to 48°45’551”N/04°05’557”W, (ii) 48°45’474”N/04°05’581”W to 48°45’500”N/04°05’537”W, (iii) 48°45’520”N/04°05’613”W to 48°45’547”N/04°05’559”W and (iv) 48°45’573”N/4°05’574”W to 48°45’501”N/4°05’625”W. All sediment samples were soaked and stirred in fresh water, to cause the release of the meiofauna from the sediment grains by osmotic shock. Subsequently, the meiofauna was extracted by decantation through a 45 μm mesh net (mermaid bra), transferred to Petri dishes with filtered sea water and sorted out under a stereomicroscope. Loricifera specimens were then narcotized for 10 min by adding drops of a 7% MgCl_2_ solution until reaching a 1:1 mixture in filtered sea water, and fixed for 1 h at room temperature in 4% paraformaldehyde (PFA). Afterwards, all specimens were washed 3 × 15 min in PBS containing 0.1% sodium azide (NaN_3_) and stored at 4°C. For muscle visualization, several stored specimens were washed 3 × 15 min in 0.1 mol l^-1^ PBS and subsequently permeabilized for 24 h at room temperature in 0.1 mol l^-1^ PBS containing 5% Triton X-100 (PBT). The specimens were then stained at 4°C for 24 h in a 1:40 dilution of Alexa Fluor 488-conjugated phalloidin (Molecular Probes) in PBT. Finally, the stained samples were washed in PBS (3 × 15 min) and mounted in Vectashield (Vector Laboratories) or Fluoromount G (SouthernBiotech) antifade mounting medium on glass slides. The use of Loricifera in the laboratory does not raise any ethical issues and therefore Regional or Local Research Ethics Committee approvals are not required.

### Confocal Laser Scanning Microscopy (CLSM) and 3D reconstruction

The total number of specimens investigated was 11 adult *Nanaloricus* sp. and 2 Higgins larvae of *Armorloricus elegans*. The fluorescence preparations were analyzed with a Leica DM 5000 CS microscope equipped with a Leica DM 5000 SP5 confocal laser scanning unit. Alternatively, a Zeiss LSM700 confocal mounted on a Zeiss Axio Imager upright microscope was used to perform the analysis. The resulting image stacks were surface-rendered with the image editing software Imaris v. 7.5.0 (Bitplane AG, Zürich, Switzerland) to create the 3D models. Additionally, the specimens embedded for CLSM were also studied using a Leica DM6000 B light microscope with phase-contrast and DIC optics.

## Competing interests

The authors declare that they have no competing interests.

## Authors’ contributions

RCN collected the animals, participated in the conception of the study, carried out the experiments, coordinated and participated in the analysis, and drafted the manuscript. HR acquired funding for the materials, participated in the conception of the study, collected the animals and revised the manuscript. XB and FL collected animals and participated in data processing and analysis. RMK and MVS collected animals, participated in the conception of the study, coordinated and participated in the analysis, and helped to draft the manuscript. All authors read and approved the final manuscript.

## Supplementary Material

Additional file 1: Video 1 Motion of adult Loricifera. Click here for file

Additional file 2: Video 2 Motion of adult Loricifera. Click here for file
